# Can We Reverse this Trend? Exploring Health and Risk Behaviours of Grade 12 Cohorts of Ontario Students from 2013–2019

**DOI:** 10.3390/ijerph18063109

**Published:** 2021-03-18

**Authors:** Adam G. Cole, Rachel E. Laxer, Karen A. Patte, Scott T. Leatherdale

**Affiliations:** 1 Faculty of Health Sciences, Ontario Tech University, Oshawa, ON L1G 0C5, Canada; 2 Public Health Ontario, Toronto, ON M5G 1V2, Canada; rachel.laxer@oahpp.ca; 3 Faculty of Applied Health Sciences, Brock University, St. Catharines, ON L2S 3A1, Canada; kpatte@brocku.ca; 4 School of Public Health and Health Systems, University of Waterloo, Waterloo, ON N2L 3G1, Canada; sleatherdale@uwaterloo.ca

**Keywords:** adolescents, health risk behaviours, lifestyle, trends, prevention

## Abstract

Adolescents engage in multiple health risk behaviours that put them at risk of future chronic disease. By the time students graduate from secondary school, they may be engaging in behaviours that set them on a particular health trajectory. It is important to monitor the co-occurrence of health risk behaviours of cohorts of grade 12 students over time to highlight important areas for intervention. The purpose of this study was to examine trends in health and risk behaviours over six waves among subsequent cohorts of grade twelve students from Ontario, Canada. A total of 44,740 grade 12 students participated in the COMPASS study across the six waves (2013/14 to 2018/19), and self-reported movement (physical activity, screen time, sleep), dietary (fruit and vegetables, breakfast), and substance use (smoking, vaping, binge drinking, and cannabis use) behaviours. Over 91.0% of students reported engaging in three or more health risk behaviours, with increases in the number of students reporting inadequate sleep, not eating breakfast on every school day, and vaping over time. Although modest, the wave 6 cohort reported slightly more risk behaviours compared with the wave 1 cohort, highlighting the importance of multidimensional health promotion strategies across multiple settings.

## 1. Introduction

The transition from adolescence to adulthood is marked by increased autonomy and the establishment of various health behaviours. Major life transitions, such as graduating from secondary school, are often characterized by changes to a person’s social and physical environments that can encourage or discourage healthy behaviours. Adolescents commonly engage in behaviours that place them at an increased risk for chronic disease, such as physical inactivity, sedentary behaviour, inadequate fruit and vegetable consumption, and substance use [1,2]. To improve upon and encourage healthier behaviours, Canada and other countries have developed various guidelines and recommendations (e.g., Canadian 24-h Movement Guidelines [3], Canada’s Food Guide [4], Low-risk Drinking Guidelines [5], Lower-risk Cannabis Use Guidelines [6], World Health Organization’s Movement Guidelines [7]).

Many adolescents engage in more than one risk behaviour, amplifying their risk of chronic disease [2]. For example, European adolescents reporting high amounts of internet use also reported poor sleeping habits, tobacco use, poor nutrition, and low levels of physical activity [8]. Other studies have explored multiple risk behaviours (e.g., inadequate fruit, vegetable, and breakfast consumption, physical inactivity, smoking, alcohol and cannabis use) and found that few adolescents reported no risk behaviours; rather, most reported more than two risk behaviours, with the number of co-occurring behaviours increasing with age [1,9,10,11,12].

The transition out of secondary school for adolescents is an important developmental milestone where there is potential for behaviour change, setting them on a healthier trajectory. Individuals engaging in risk behaviours, such as alcohol or cannabis use, during their final year of secondary school are likely to continue and increase their frequency of use during their first year of college [13]. They may also begin engaging in additional risky behaviours concurrently. This might be explained by an increase in autonomy, decreased adult supervision, and the availability and opportunity to engage in these behaviours more freely [14].

Although many studies have explored the health risk behaviours of students, to our knowledge few have examined trends across time. Monitoring the co-occurrence of health risk behaviours of grade 12 students may highlight important areas for intervention, since interventions addressing multiple risk behaviours tend to be a more effective approach [15]. It is equally important to monitor changes in co-occurring behaviours over time as national-level policies change and in light of changing public health guidelines and recommendations. For example, given the recent release of Canada’s 24-h Movement Guidelines and updated Food Guide recommendations, as well as changes to substance use policies (e.g., national legalization of cannabis, increased access to e-cigarettes and vaping devices, the sale of alcohol in grocery stores across Ontario), it is important to identify whether these policy changes have impacted the patterns of health-risk behaviour co-occurrence over time. Therefore, the objective of this study was to examine trends in health and risk behaviours over six waves among subsequent cohorts of grade 12 students in Ontario, Canada.

## 2. Materials and Methods

### 2.1. Sample Selection

This study used repeat cross-sectional data from a non-probability sample of grade 12 students in Ontario participating in the COMPASS study between 2013/14 (wave 1) and 2018/19 (wave 6). COMPASS is a nine-year (2012–2021) school-based, prospective cohort study designed to evaluate the impact of changes to programs, policies, and the built environment on multiple youth health behaviours and outcomes over time [16]. Between 2013/14 and 2018/19, public and private schools that used active-information passive-consent parental permission protocols (passive consent) were purposefully sampled from Ontario [17]. The use of passive consent limits bias that results from student non-response and non-participation common in studies of youth risk behaviours that use active consent procedures that require participants to return a permission form signed by their parent/guardian [18,19]. Additional details regarding the recruitment methods of the COMPASS study can be found in print [16] or online (www.compass.uwaterloo.ca, accessed on 17 March 2021). The University of Waterloo Office of Research Ethics (ORE 30118) and participating school board ethics committees approved all procedures.

Within the COMPASS host study, the sample of grade 12 students (n) and schools (N) by wave were as follows: wave 1 (2013/14): n = 9197, N = 79; wave 2 (2014/15): n = 8155, N = 78; wave 3 (2015/16): n = 7867, N = 72; wave 4 (2016/17): n = 6968, N = 68; wave 5 (2017/18): n = 6392, N = 61; and wave 6 (2018/19): n = 6161, N = 61. Students with missing responses for gender, age, and ethnicity, and with an age under 16 years were excluded from the sample (n = 898), leaving a total of 44,740 grade 12 students across the 6 years. Across years, student participation rates were generally high (>75%) and participant demographics were similar. Demographic characteristics by wave were as follows: wave 1: 48.7% female and 79.7% white; wave 2: 49.6% female and 78.4% white; wave 3: 48.2% female and 76.6% white; wave 4: 49.2% female and 77.2% white; wave 5: 48.6% female and 74.6% white; and wave 6: 48.9% female and 73.4% white.

### 2.2. Measures

Data were collected annually from students using the COMPASS questionnaire (Cq), a paper-based survey completed during class time between October and June every year. In addition to information on sociodemographic characteristics (grade, gender), the Cq collects information on a variety of health risk behaviours using items consistent with other school-based research in Canada and with demonstrated reliability and validity [20,21].

#### 2.2.1. Movement Behaviours

Students indicated the number of minutes of moderate-to-vigorous physical activity (MVPA) they did on each of the last 7 days. Consistent with Canada’s 24-h Movement Guidelines for children and youth [3], those who reported achieving at least 60 min of MVPA per day over each of the last 7 days had adequate physical activity levels, while all others had inadequate physical activity levels. While the Canadian Society of Exercise Physiologists recommends three days of strength training per week, this indicator was not included as a stand-alone measure and would have been considered as part of the MVPA.

Students reported how much time per day they usually spent sleeping. Consistent with Canada’s 24-h Movement Guidelines for children and youth [3], those who reported getting more than 8 h of sleep in a usual night had adequate sleep levels, while all others had inadequate sleep levels. It is important to note that response options on the Cq were in hour (0–9) and 15-min intervals (0, 15, 30, 45) so it is not possible to identify those that exceeded the guideline.

Students reported how much time per day they usually spent doing each of three screen-based sedentary activities: watching/streaming TV shows or movies, playing video/computer games, and surfing the internet. Consistent with Canada’s 24-h Movement Guidelines for children and youth [3], those who reported spending less than 2 h total in a usual day on these activities were considered as meeting the recreational screen time guideline, while respondents reporting 2 or more hours per day were considered as exceeding the guideline.

#### 2.2.2. Dietary Behaviours

Students reported the number of servings of vegetables and fruits they had the previous day. The Cq included examples and images of serving sizes of vegetables and fruits from the 2007–2019 Canada’s Food Guide (which has since been updated). Consistent with the previous Canada’s Food Guide recommendations for youth [4], female students who reported eating 7 or more servings and male students who reported eating 8 or more servings of vegetables and fruits the previous day had adequate fruit and vegetable consumption; all others had inadequate fruit and vegetable consumption.

Students also reported the number of days in a usual school week (Monday to Friday) that they ate breakfast. Students that reported eating breakfast 5 days in a usual school week had adequate breakfast consumption while all others had inadequate breakfast consumption.

#### 2.2.3. Substance Use

Students reported whether they smoked one or more cigarettes, smoked cigarillos or little cigars (plain or flavoured), used smokeless tobacco (chewing tobacco, pinch, snuff, or snus), or smoked hookah (water-pipe) in the last 30 days. They also reported whether they used e-cigarettes (herein called vapes). The question for vape use included the following explanation: “electronic cigarettes that look like cigarettes/cigars, but produce vapour instead of smoke.”

Students reported how often they had 5 drinks of alcohol or more on one occasion (binge drinking) in the last 12 months. In line with other national surveys, students were instructed that a drink means 1 regular sized bottle, can, or draft of beer; 1 glass of wine; 1 bottle of cooler; 1 shot of liquor (rum, whisky, etc.); or 1 mixed drink (1 shot of liquor with pop, juice, energy drink). Those who reported having 5 drinks of alcohol once a month or more frequently were considered monthly binge drinkers. Similarly, students reported how often they used marijuana or cannabis (a joint, pot, weed, hash) in the last 12 months. Monthly cannabis use was determined based on reports of once a month or more frequent use.

### 2.3. Analysis

Statistical analyses were conducted using SAS software, version 9.4 (SAS Institute Inc., Cary, NC, USA) [22]. We examined the prevalence of each health behaviour for each year and the mean number of risk factors that students reported at each year. Trends in health behaviours were examined by gender. Analysis of variance (ANOVA) tested for significant differences in the mean number of co-occurring risk behaviours over time for each gender and overall. Statistical significance was set at *p* < 0.05.

## 3. Results

**Figure 1:**
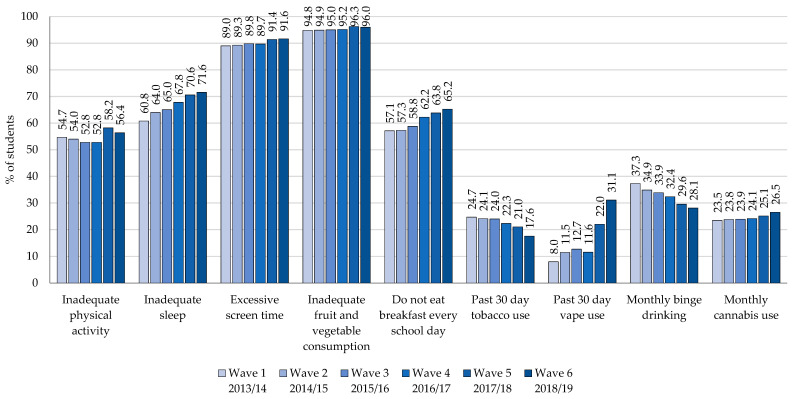
Percentage of grade 12 students (n = 44,740) engaging in health risk behaviours by wave, 2013-19 COMPASS study.

Figure 1 presents the percentage of grade 12 students engaging in health risk behaviours by survey wave, and Table 1 presents the results by gender. With respect to movement behaviours, across waves >80% of students reported exceeding the sedentary behaviour guideline (more than 2 h of screen-time) on a usual day with only slight changes from year-to-year. Slightly more than half of students reported MVPA levels that fell short of Canada’s guidelines, and this remained relatively consistent for both male and female students over the survey waves. In contrast, the number of students reporting inadequate sleep increased by 17% for males and 18% for females between wave 1 and 6.

With respect to dietary behaviours, across waves >90% of students reported inadequate fruit and vegetable consumption according to the 2007–2019 Canada’s Food Guide recommendations with only slight changes from year-to-year. More than half of students reported not eating breakfast every school day, and this percentage increased between wave 1 and 6, particularly among female students.

With respect to substance use behaviours, the prevalence of past 30-day cigarette smoking was relatively consistent before decreasing in wave 6, at which point 32% fewer male students and 22% fewer female students reported smoking cigarettes in the last 30 days (relative to wave 1). Similarly, the prevalence of monthly binge drinking decreased across waves among both male (26%) and female (21%) students. In contrast, the prevalence of past 30-day vaping increased dramatically, particularly during the most recent years. Between wave 1 and 6, past 30-day vaping increased by 216% among male students and 416% among female students. Monthly cannabis use also increased among both male (8%) and female (20%) students.

**Figure 2:**
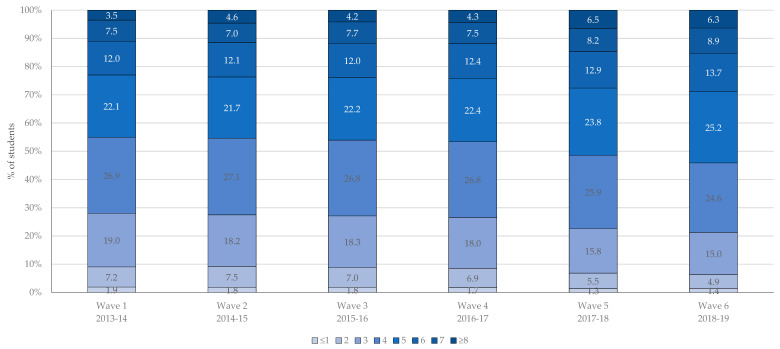
Prevalence of co-occurring health risk behaviours among grade 12 students (n = 44,740) by wave, 2013-19 COMPASS study.

As shown in Table 2, students reported an average of 4.4 co-occurring health risk behaviours in wave 1; the mean increased to 4.8 co-occurring health risk behaviours by wave 6. Across waves, the mean number of co-occurring health risk behaviours increased among both male and female students. Students reported an average of 2 (out of a possible 3) co-occurring risk behaviours within the movement subcategory, 1.5 (out of a possible 2) co-occurring risk behaviours within the dietary subcategory, and 1 (out of a possible 4) co-occurring risk behaviours within the substance use subcategory. The mean number of co-occurring risk behaviours increased significantly among male and female students within the three subcategories of behaviours (movement, dietary, and substance use). As shown in Figure 2, less than 10% of students reported 2 or fewer co-occurring health risk behaviours and this percentage decreased between wave 1 and 6. In contrast, more than 10% of students reported 7 or more co-occurring health risk behaviours and this percentage increased between wave 1 and 6.

## 4. Discussion

This study used data collected annually from a large sample of Ontario grade 12 students participating in six waves of the COMPASS study from 2013/14 to 2018/2019. Most students reported engaging in multiple health risk behaviours, placing them at risk of various chronic diseases in the future. The progressive cohorts were engaging in slightly more risk behaviours over time; though modest, the risk profiles were trending in a worsening trajectory. Over the study period, the number of health risk behaviours that students reported gradually increased from an average of 4.4 co-occurring risk behaviours to an average of 4.8. Our results indicate that not only were there variations in the prevalence of health risk behaviours over time, but there were differences across gender.

Adequate physical activity, limiting sedentary time, and a diet high in fruits and vegetables are recommended for chronic disease prevention [23,24,25,26]. Across all waves, the majority of students consistently reported exceeding screen-based sedentary time guidelines, inadequate levels of physical activity, and not meeting fruit and vegetable recommendations. In the absence of interventions, these behaviours will only continue to worsen and more students will fail to meet recommendations. Knowing that physical activity levels tend to decline through adolescence, especially among females [27,28], suggests that addressing these behaviours earlier might be one effective method for prevention. For example, one study found that community sport participation and parental support and encouragement were associated with an increase in the proportion of grade 12 students meeting physical activity and screen time recommendations, after not meeting the guidelines in grade nine [27]. Screen-based sedentary time is ubiquitous and therefore it is important to identify healthy ways to manage screen time among adolescents. For example, recommendations on healthy media might also include restrictions on electronic devices during the school day. Our data also indicate that most (94–96%) grade 12 students across all data collection waves were not meeting recommendations for fruit and vegetable consumption. Population-level solutions, such as school food and beverage programs to make fruits and vegetables more accessible and available, might be most impactful.

Over time, fewer students reported meeting sleep recommendations or eating breakfast every day during the school week. This trend is consistent with previous studies that reported a steep decline in sleep duration over time [29,30,31], with declines up to 0.75 min per night per year [32], alongside increased reports of tiredness and difficulties sleeping. In another study using COMPASS data, average sleep duration dropped approximately eight minutes across three study waves (7.47 to 7.34 h per night), with fewer students meeting the guideline of 8–10 h per night in 2015/16 (45.3%) compared to 2013/14 (50.3%) [31]. This growth in insufficient sleep time among adolescents might be explained by excessive screen time (e.g., excessive light exposure, over-stimulation, and/or displacement), dietary behaviours (e.g., high caffeine consumption, sugar-sweetened beverage consumption, poor diet quality), social activities, other obligations (e.g., homework or extracurricular activities), physical inactivity, distress, or substance use [33,34,35,36]. Adequate sleep is an important contributing factor to both physical and mental health among adolescents [37]. Among the movement behaviours, adherence to the sleep guidelines has emerged as the most consistent predictor of depressive symptoms and psychosocial wellbeing among adolescents [38,39,40] and appears to mediate the effect of school pressures, physical activity, and screen time on psychological distress [41]. Poor sleep could also impede physical activity levels, resistance to peer influences [42], and engagement in substance use [43]. Later school start times have been recommended to improve adolescent sleep time, with even modest delays demonstrating benefits for youth sleep durations [44,45] and breakfast consumption [46]. Despite the fact that many schools have breakfast programs, our results are consistent with reports that many students still skip breakfast [47] and the prevalence of this behaviour appears to be increasing. Breakfast skipping has been linked to reduced daily dietary intakes [48] and increased cardiometabolic risk [49]. Furthermore, those who skip breakfast may engage in irregular eating patterns (such as late night snacking), which can have a negative impact on sleep quality [36]. A bidirectional association may exist between diet and sleep, and future research should explore this association and whether improvements in one behaviour have a positive effect on the other

Our results also indicate changes in substance use behaviours across the grade 12 cohorts. The largest relative change to the health behaviours investigated in this study was vaping, with a 461% relative increase among female students and a 216% relative increase among male students from wave 1 to wave 6. Consistent with other data [50,51,52], the prevalence of past 30-day tobacco use decreased across waves of grade 12 students, particularly in the most recent survey years coinciding with major increases in youth vaping [53]. This rise in youth vaping is alarming, and aligns with the evolution of the vaping market and tobacco companies’ heavy advertising of their e-cigarette brands in Canada, following the legalization of e-cigarettes with nicotine [54]. Decreasing exposure to marketing through provincial restrictions would be an effective strategy to reduce vaping and reverse the current trend [54]. Importantly, the prevalence of monthly binge drinking decreased, coinciding with an increase in the prevalence of cannabis use, with almost one quarter of female students and one-third of male students reporting monthly cannabis use. Cannabis use during adolescence can lead to functional and structural changes to the brain, possibly resulting in cannabis dependence and substance use disorders [55]. Moreover, cannabis use might lead to the initiation and maintenance of tobacco smoking and vaping, and has been linked to the use of alcohol and heavy drinking in adulthood [33,55]. There is a need to identify and implement policies and programs to reduce substance use among youth.

In addition to individual trends, a unique result of this study was in identifying changes to the mean number of co-occurring behaviours over time. Our results indicate that the mean number of co-occurring behaviours increased over time, both overall and within subcategories of behaviours across both genders. In this study, male students engaged in a higher number of mean co-occurring risk behaviours compared to female students and the difference was largely driven by differences in substance use. Understanding the mechanism by which, and why these behaviours co-occur might be the first step in identifying the appropriate prevention strategies. For example, among the students participating in this study, the changes in trends were largely driven by substance use given that so many students reported inadequate fruit and vegetable consumption and excessive screen time consistently across waves. Some of these behavioural patterns might also be explained by sleep duration, since low sleep duration increases risk for engaging in all other risk behaviours and sleep duration decreased across waves [42,43]. Despite the concerning number of co-occurring behaviours, it is normative to engage in risk behaviours during adolescence, and is therefore important to build harm reduction practices to prevent harmful and worsening trajectories as students graduate from high school.

These findings have important implications for policy and practice. Given that more than 90% of grade 12 students in our sample engaged in three or more risk behaviours and that they spend a large part of their day at school, interventions should target these critical, teachable times in both elementary and secondary schools. Graduating from secondary school is an important life transition when health beliefs and behaviours may be amenable to change and when established lifestyle habits might persist into later life and affect long-term health and social outcomes. The risk profiles of the majority of students in this study, however, indicate that changes might be required beyond the mandate of schools, at a broader societal level, including changes to healthy built environments and changes to policies. Additionally, given the multiple transitions that happen after graduating secondary school and the evidence that risk behaviours increase in frequency following graduation [13], interventions should target postsecondary institutions and workplaces. Future research should investigate the progression of health risk and co-occurring behaviours during adolescence to identify important stages when risk behaviours change that could be targeted. Finally, continued monitoring of multiple health risk behaviours is necessary to evaluate how changes to provincial and national guidelines, recommendations, and policies influence multiple health behaviours. Future research might explore the use of substitution models to examine how changes in particular behaviours, such as use of screen time, might affect other behaviours, such as sleep or physical activity.

This study is not without limitations. COMPASS uses a non-probability, school-based sample that is not necessarily representative of all youth in Ontario. However, the use of passive consent protocols limits the bias that results from student non-response and non-participation that are common in active consent surveys [18,19]. Second, the use of self-reported measures of all health risk behaviours introduces a risk of social-desirability bias and recall bias. However, students were assured that all responses would remain anonymous and the measures in the Cq have demonstrated reliability and validity [20,21] and are consistent with other surveillance measures in Canada. Any bias introduced would likely result in an underestimation of substance use and overestimation of movement and dietary behaviours, thereby suggesting that youth might have a higher number of co-occurring behaviours [56]. Third, the screen time measures used in this study did not specify recreational use as per the 24-h Movement Guidelines, and did not account for potential multitasking or intermittent patterns of use; therefore, it is possible that students either over-reported or under-reported their total sedentary screen time. Finally, the measure of physical activity used in this study was based on accumulated MVPA and did not include light physical activity or resistance exercise/strength training. Strengths of this study include the use of repeated waves of data for multiple health risk behaviours to look at trends over time, and the large sample of students included. This study is the first of its kind to explore patterns of reporting of common health risk behaviours in grade 12 students across different time periods.

## 5. Conclusions

These data indicate that health risk behaviours are common among grade 12 students in Ontario, Canada, which has important implications for their future health and wellbeing. The prevalence of many health risk behaviours among subsequent grade 12 cohorts either stayed the same or increased and most students engaged in multiple health risk behaviours, highlighting the importance of multidimensional health promotion strategies targeting adolescents. Additional monitoring and research is necessary to identify effective approaches to reduce health risk behaviours among youth.

## Figures and Tables

**Table 1:** Percentage of grade 12 students (n = 44,740) engaging in health risk behaviours by gender and wave, 2013-19 COMPASS study.

Gender	Data Collection Wave						
	Wave 1 2013/14 n (%)	Wave 2 2014/15 n (%)	Wave 3 2015/16 n (%)	Wave 4 2016/17 n (%)	Wave 5 2017/18 n (%)	Wave 6 2018/19 n (%)	% Change (Relative) from Wave 1 to 6
**Movement behaviours**							
Inadequate physical activity							
Female	2744 (62.2)	2420 (60.9)	2285 (61.2)	1991 (59.5)	2020 (65.9)	1922 (64.7)	+4.0%
Male	2179 (47.4)	1876 (47.1)	1755 (44.7)	1578 (46.2)	1632 (50.8)	1490 (48.4)	+2.0%
Inadequate sleep duration							
Female	2777 (62.2)	2668 (66.2)	2521 (66.6)	2373 (69.5)	2240 (72.5)	2204 (73.4)	+18.1%
Male	2790 (59.5)	2533 (61.9)	2574 (63.5)	2325 (66.2)	2240 (68.8)	2176 (69.8)	+17.4%
Excessive screen-based sedentary time							
Female	3800 (85.1)	3495 (86.7)	3322 (87.8)	3000 (87.9)	2741 (88.7)	2685 (89.4)	+5.1%
Male	4354 (92.8)	3759 (91.8)	3713 (91.7)	3216 (91.5)	3062 (94.0)	2924 (93.8)	+1.1%
**Dietary behaviours**							
Inadequate fruit and vegetable consumption							
Female	4134 (94.0)	3779 (95.1)	3532 (94.7)	3195 (95.2)	2923 (95.8)	2850 (96.3)	+2.4%
Male	4348 (95.6)	3732 (94.7)	3710 (95.3)	3228 (95.2)	3046 (96.7)	2882 (95.7)	+0.1%
Do not eat breakfast every school day							
Female	2570 (58.2)	2366 (59.1)	2295 (61.3)	2187 (64.7)	2052 (67.0)	2040 (68.8)	+18.1%
Male	2578 (56.1)	2224 (55.4)	2227 (56.5)	2066 (59.9)	1937 (60.8)	1891 (61.8)	+10.3%
**Substance use behaviours**							
Past 30 day tobacco use							
Female	797 (17.8)	724 (17.9)	677 (17.9)	593 (17.5)	493 (16.0)	415 (13.9)	−22.1%
Male	1475 (31.3)	1240 (30.2)	1210 (29.7)	940 (27.0)	838 (25.9)	655 (21.1)	−32.5%
Past 30 day vape use							
Female	219 (4.9)	322 (8.0)	302 (8.0)	266 (7.8)	488 (15.7)	827 (27.4)	+461.2%
Male	517 (11.0)	616 (15.0)	694 (17.0)	541 (15.3)	918 (27.9)	1090 (34.6)	+216.4%
Monthly binge drinking							
Female	1467 (32.9)	1230 (30.5)	1092 (28.9)	1023 (29.9)	804 (25.9)	773 (25.7)	−21.7%
Male	1954 (41.6)	1599 (39.1)	1563 (38.6)	1224 (34.7)	1080 (33.0)	(952) 30.4	−26.9%
Monthly cannabis use							
Female	813 (18.4)	735 (18.5)	667 (17.9)	664 (19.6)	660 (21.5)	661 (22.2)	+20.5%
Male	1299 (28.4)	1161 (29.1)	1165 (29.6)	977 (28.6)	919 (28.7)	946 (31.0)	+8.0%

**Table 2:** Mean number of co-occurring health risk behaviours among grade 12 students (n = 44,740) by wave, gender, and risk behaviour category, 2013-19 COMPASS study.

Risk Behaviour Category and Gender	Data Collection Wave						ANOVA
	Wave 1 2013/14 Mean (stdev)	Wave 2 2014/15 Mean (stdev)	Wave 3 2015/16 Mean (stdev)	Wave 4 2016/17 Mean (stdev)	Wave 5 2017/18 Mean (stdev)	Wave 6 2018/19 Mean (stdev)	
Overall	4.4 (1.6)	4.5 (1.6)	4.5 (1.6)	4.5 (1.6)	4.7 (1.6)	4.8 (1.6)	F = 51.8, *p* < 0.001
Female	4.3 (1.5)	4.4 (1.5)	4.4 (1.5)	4.5 (1.5)	4.6 (1.6)	4.8 (1.5)	F = 44.2, *p* < 0.001
Male	4.6 (1.6)	4.6 (1.7)	4.6 (1.7)	4.5 (1.7)	4.8 (1.7)	4.8 (1.7)	F = 15.2, *p* < 0.001
Movement behaviours (maximum 3)	2.0 (0.8)	2.1 (0.8)	2.1 (0.8)	2.1 (0.8)	2.2 (0.7)	2.2 (0.7)	F = 57.5, *p* < 0.001
Female	2.1 (0.8)	2.1 (0.8)	2.1 (0.8)	2.2 (0.8)	2.3 (0.7)	2.3 (0.7)	F = 34.5, *p* < 0.001
Male	2.0 (0.8)	2.0 (0.8)	2.0 (0.8)	2.0 (0.8)	2.1 (0.8)	2.1 (0.7)	F = 25.5, *p* < 0.001
Dietary behaviours (maximum 2)	1.5 (0.6)	1.5 (0.6)	1.5 (0.6)	1.6 (0.6)	1.6 (0.5)	1.6 (0.5)	F = 31.9, *p* < 0.001
Female	1.5 (0.6)	1.5 (0.6)	1.5 (0.6)	1.6 (0.6)	1.6 (0.5)	1.6 (0.5)	F = 26.9, *p* < 0.001
Male	1.5 (0.6)	1.5 (0.6)	1.5 (0.6)	1.5 (0.6)	1.5 (0.6)	1.5 (0.6)	F = 9.2, *p* < 0.001
Substance use behaviours (maximum 4)	0.9 (1.2)	0.9 (1.2)	0.9 (1.2)	0.9 (1.2)	1.0 (1.3)	1.0 (1.3)	F = 8.8, *p* < 0.001
Female	0.7 (1.0)	0.7 (1.1)	0.7 (1.1)	0.7 (1.1)	0.8 (1.1)	0.9 (1.2)	F = 10.3, *p* < 0.001
Male	1.1 (1.3)	1.1 (1.3)	1.1 (1.3)	1.0 (1.3)	1.1 (1.4)	1.2 (1.4)	F = 3.5, *p* < 0.01

## Data Availability

Data from the COMPASS study are available following approval of the COMPASS data usage application form from the following website: https://uwaterloo.ca/compass-system/information-researchers (accessed on 17 March 2021).
